# Secular trends in the incidence of major depressive disorder and dysthymia in China from 1990 to 2019

**DOI:** 10.1186/s12889-023-17025-4

**Published:** 2023-11-06

**Authors:** Ming Li, Wenlong Gao, Yuqi Zhang, Qiuxia Luo, Yuanyuan Xiang, Kai Bao, Noha Zaki

**Affiliations:** Institute of Epidemiology and Health Statistics, School of Public Health, Lanzhou University, Tianshui Southern Road 222, Lanzhou, Gansu P. R. China

**Keywords:** Depressive disorders, Incidence, Dysthymia, China, Joinpoint regression, Age–period–cohort analysis

## Abstract

**Background:**

Depression is increasingly recognized as a worldwide serious, public health concern. A better understanding of depression is important for advancing its management and learning the difference between major depressive disorder (MDD) and dysthymia. Our aim is to conduct a concurrent analysis of the trends of both MDD and dysthymia in China.

**Methods:**

The data on depression from 1990 to 2019 were collected from the Global Burden of Disease Study 2019 (GBD 2019). To determine the average annual percent changes (AAPC) and relative risks (RRs), joinpoint regression and the age-period-cohort models were employed, respectively.

**Results:**

The incidence number of MDD and dysthymia continuously increased in China from 1990 to 2019, however, the age-standardized rates (ASR) had a decreasing trend in both men and women. The results from joinpoint regression showed that a declining trend was presented in young people (< 50 years) but an increased trend in the elderly (≥ 50 years) both in men and women, during 1990–2019. Age is the most influential factor for MDD and dysthymia. Age RRs for MDD incidence had an overall increasing trend with age. Period RR in MDD presented a U-shaped pattern, while Cohort RRs presented an inverted U-shaped pattern. On the other hand, RRs in dysthymia for period and cohort effects had no statistical significance, only the age effect presented an inverted U-shaped pattern.

**Conclusions:**

The disparities in trends observed between MDD and dysthymia during the period of 1990–2019 indicated the significance of distinguishing between these two disorders. The age, period and cohort effects all had a greater impact on MDD than on dysthymia, and age effects presented different influential patterns in these two. To alleviate the burden of depressive disorders in China, proactive measures need to be implemented, with particular attention to the elderly population.

**Supplementary Information:**

The online version contains supplementary material available at 10.1186/s12889-023-17025-4.

## Introduction

Mental disorders are recognized as the leading cause of global disease burden [1], and depressive disorders accounted for the highest proportion of disability-adjusted life-years (DALYs) among all mental illnesses according to the Global Burden of Diseases, Injuries, and Risk Factors Study (GBD) 2019 [2]. This high burden was throughout the entire lifespan of different gender [2]. More importantly, previous studies indicated that although many effective interventions were applied, no reduction of global prevalence and burden had been observed since 1990 [3]. According to the GBD 2019 database, there are two subtypes of depressive disorders: major depressive disorder (MDD) and dysthymia. It is worth noting that while MDD has received more research attention, dysthymia has been frequently overlooked clinically, due to its milder symptoms than other chronic mental disorders [4]. However, there is no denying that it has an impact on body function and long-term outcomes. Specifically, longitudinal researches have showed that dysthymia may increase subsequent difficulties in activities of daily living, co-morbidity and physical frailty among the elderly population [4–6].

MDD is characterized by more severe symptoms compared to dysthymia. It can have a chronic intermittent episode or a short-term episode course, whereas dysthymia typically follows a long course [7]. There are varying terms for chronic depressive disorders in different diagnostic criterion [8, 9]. For example, while the concept of chronic depressive disorders exists in both the International Classification of Diseases (ICD-10) and the fifth edition of the Diagnostic and Statistical Manual of Mental Disorders (DSM-5), the ICD-10 designates this condition as “dysthymia” whereas the DSM-5 refers to it as "persistent depressive disorder", encompassing various forms of chronic depressive conditions. These differences pose a challenge of synthesizing and comprehending the literature, as well as conducting comparisons across various studies. It may be useful that conceptualize depressive disorders from the dimensions of severity and longitudinal trends. Thus, we try to analyze the temporal trends of MDD and dysthymia at the same time.

China has the second largest population in the world and also faces the challenge of an increasing burden of mental disorders, particularly depressive disorders [10, 11]. The prevalence of mental health in China was lower than many other nations, while Australasia, Tropical Latin America, and high-income North America had the highest prevalence worldwide. The prevalence rate of mental illness in China was reported as 9.3% (95% confidence interval (CI): 5.4-13.3%) in China [10]. In 2017, depression accounted for 2.3% of all DALYs in China, of which major depression accounted for 1.55% and dysthymia accounted for 0.75% [12]. Despite these challenges, the treatment rates of depressive disorders in China have been reported to be very low, with only a small number of individuals receiving sufficient treatment in 2019 [11]. In addition, few studies have reported the epidemiology of dysthymia in China, and even fewer about its temporal trends.

The GBD 2019 Study estimated the disease burden of depressive disorders by age, sex, year, and location with a standardized methodology, providing national-level data allows for analysis of incidence trends [12]. It’s important to note that many studies on the time trend or age trend often don’t account for the confounding effect of cohort. To account for cohort effects properly, it is necessary to establish and compare multiple cohorts over a period of time. However, a typical longitudinal study only focuses on a single cohort. In the case of the two previous studies mentioned, they did not establish multiple cohorts or subdivide a single cohort, thus failing to consider the cohort effect [13, 14]. Effective control of cohort effects can enhance cross-population comparability in research findings [15, 16]. By employing an age-period-cohort model, our study aimed to describe the secular trend of MDD and dysthymia. This approach can contribute to understanding depression from a historical perspective and may provide valuable insights for informing prevention policies.

## Methods

### Data source

The incidence data of depressive disorder were obtained from the Global Burden of Disease 2019 study, which is readily available to the public [17]. The GBD 2019 study estimated the burden of the vast majority of diseases and their risk factors in most countries and territories of the world from 1990 to 2019 [18–20]. The primary data on mental illness of GBD 2019 were mainly from published literature in electronic databases (including PsycINFO, PubMed and Embase), at the same time, supplemented by the gray literature, and expert consultation [2]. The sources of data on depression in China primarily include peer-reviewed literature and reports that have been publicly published, large-scale population and health surveys conducted within China, publicly available government data, and monitoring data from the Chinese Center for Disease Control and Prevention [2, 20]. The input sources for data on corresponding diseases in respective regions can be accessed through relevant websites, and the associated code used to synthesize the final data can also be viewed [21, 22].

Depressive disorders were defined based on the Diagnostic and Statistical Manual of Mental Disorders Fourth Edition, Text Revision (DSM-IV-TR) and International Classification of Diseases and Related Health Problems 10th Revision (ICD-10) in GBD 2019. Depressive disorders were divided into major depressive disorder (MDD) and dysthymia. MDD is defined as the presence of at least one major depressive episode, which is the experience of either depressed mood or loss of interest almost every day, and at least last two weeks, while dysthymia is denoted as the experience of chronically depressed mood for most of the day, for at least two years (or at least one year for children and adolescents).

The adjusted estimates were modeled using a Bayesian meta-regression tool that aggregates data from different sources to get internally consistent estimates for incidence, by gender, age, year and location. Previous articles have provided more detailed descriptions of the methodologies of the mental disorder part of GBD 2019 [18]. The GBD 2019 global age-standard population was used to calculate the age-standardized incidence rate and its uncertainty intervals (UIs).

### Statistics analysis

#### Joinpoint regression analysis

Like the ordinary least squares regression method, the joinpoint program was used to find the best-fit line through time. However, the joinpoint program was fitted with multi-segmented lines to obtain a better fitting than one line or less segmented line [23]. Each joinpoint means a statistically significant change in trend. For each segment, the natural logarithm of the incidence rate was fitted to a straight line as a function of the calendar year, and then its estimated annual percent change (APC) and its 95% CI were obtained [24]. The APC represents the slope of each line segment describing both the direction and gradient. The average APC (AAPC) was obtained by assuming that there was only one segment (one line) throughout the entire study period [25].

#### Age–period–cohort model

When examining the changing trends of disease rates, conventional analysis methods typically calculate rates at different time points. However, these methods face challenges in distinguishing the effects of age, period, and cohort, known as the identification problem in the age-period-cohort model [26, 27]. This problem arises due to the perfect linear dependency among these three factors: period - age = cohort. Generally, age effects are changes associated with the biological and social aging processes that are unique to individuals. From the perspective of epidemiology, age effects are usually presented as different rates of disease across age groups. The period effect represents the effect from live environment, which equally influence all age groups at a specific time. It may be caused by abundant social, cultural and economic factors, such as war, great earthquakes, famine and economic recession. Cohort effects are associated with the distinct exposure of a group of people as they together move over time, and in epidemiological definition, the group of people is the birth cohort. The age-period-cohort model could obtain the independent effect of these three factors.

Age–period–cohort model is essentially a generalized linear model. Several approaches could address the identification problem, and the intrinsic estimator (IE) method was chosen for this study [28, 29]. The intrinsic estimator method uses principal component regression techniques, and its robustness of the statistical properties has been confirmed by model validation studies [29–31]. The following interpretation may be useful for understanding the intrinsic estimator method. The ridge estimator could be used when facing independent variables that are highly collinear, intrinsic estimator can be viewed as a special case of ridge estimator as its shrinkage penalty goes to zero.

The coefficients obtained from the age-period-cohort model could be used to calculate the relative risks (RRs), which represent the effects of age, period and cohort relative to the average level of whole ages, periods and birth cohorts. The period effect provides an unbiased (controlling the effects of two other factors) description better than annual rates to assess time trends [32, 33]. The age effect estimated through the age-period-cohort model is unbiased and robust in statistics to describe age patterns of depression risk, better than age-specific rates [32, 33]. Age–period–cohort model was performed using STATA 14.0 software (Stata Corp, College Station, TX, USA).

## Results

### Descriptive analysis

**Fig. 1:**
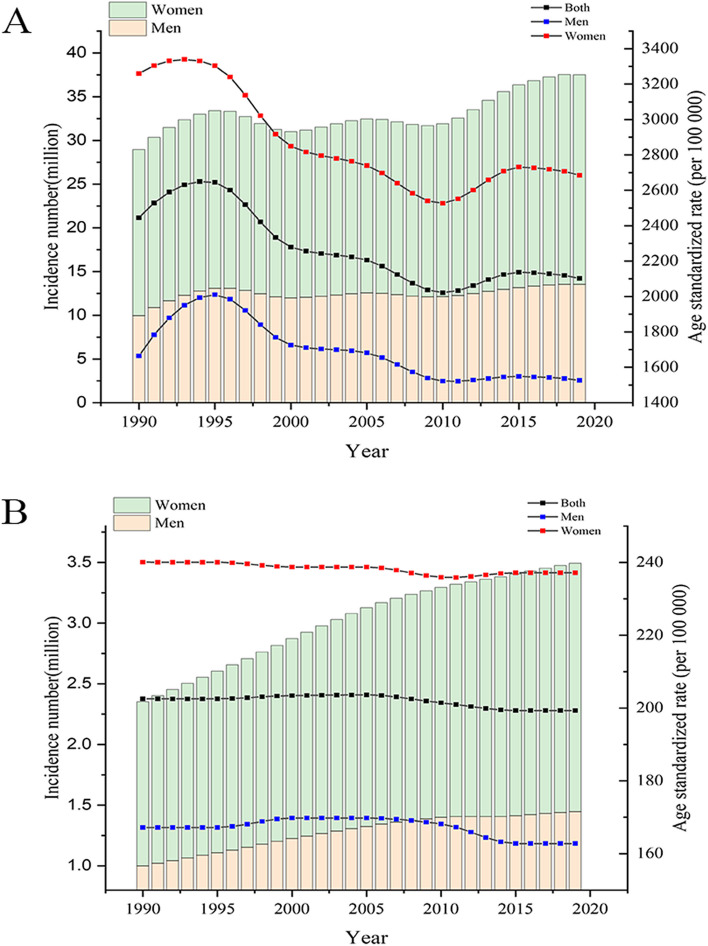
Trends in incidence number and age-standardized rate of major depressive disorders (A) and dysthymia (B). Note: The left-axis represents the number of cases, corresponding to the stacked graph; the right-axis represents the standard incidence rate, corresponding to the line graph

As shown in Fig. 1, the incidence number of MDD and dysthymia continuously increased in China from 1990 to 2019, however, the ASR had a decreasing trend, in both men and women. Their rates and numbers were higher in women than in men during the past 30 years.

In 2019, the ASRs of depressive disorders in China were 1688.81 (95% UI: 1886.11, 1505.21) per 100 000 population in men and 2922.07 (95% UI: 3271.13, 2598.4) in women. The ASRs were 1526.03 (95% UI: 1721.13, 1346.86) per 100 000 persons in men and 2684.92 (95% UI: 3023.11, 2371.05) in women for major depressive disorder and 162.79 (95% UI: 195.35, 133.35) in men and 237.16 (95% UI: 286.37, 195.67) in women for dysthymia. The incidence rates of depressive disorders increased with age in both genders.

**Fig. 2:**
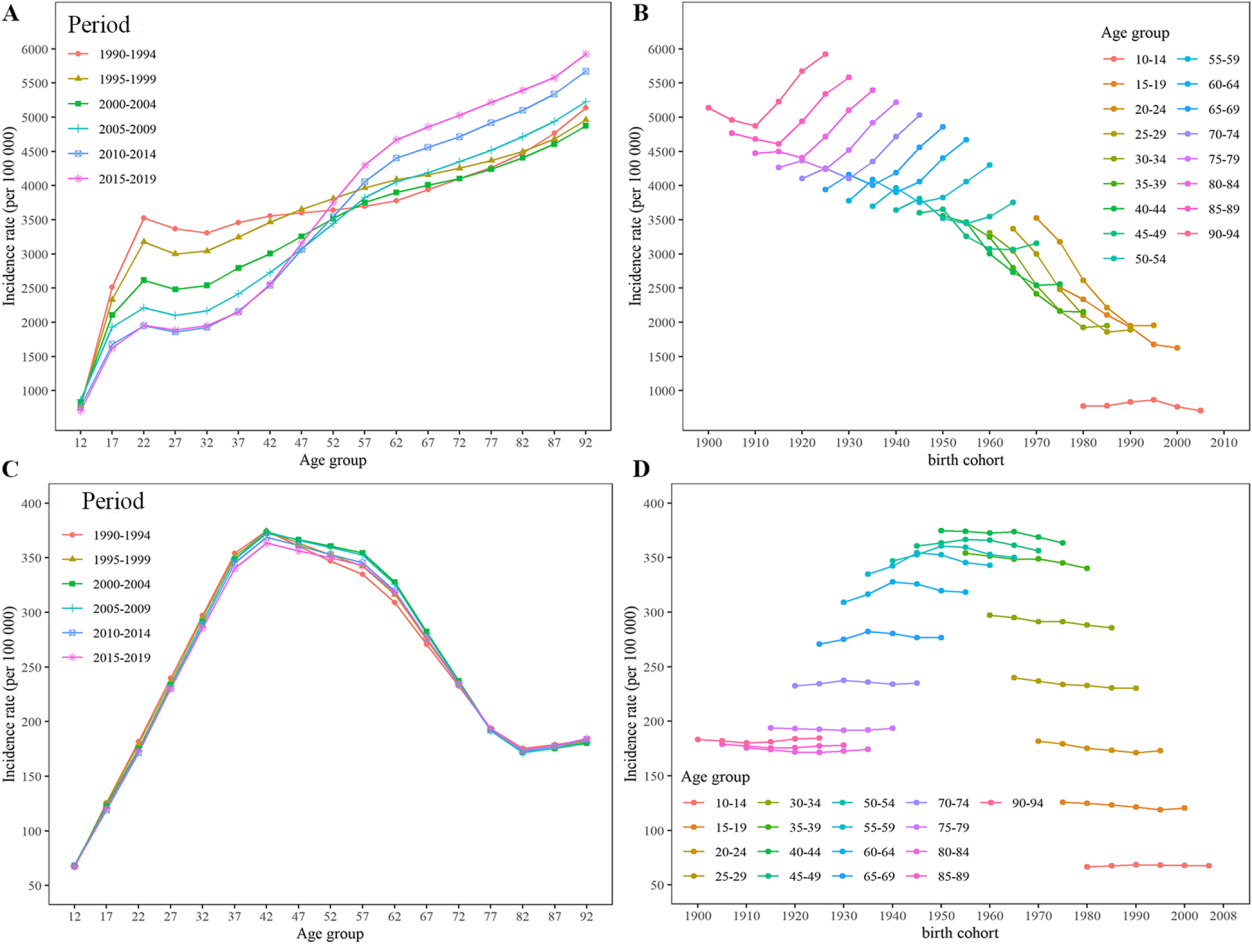
Age-specific incidence rates by period of major depressive disorder (**A**) and dysthymia (**C**); and cohort-specific incidence rates by age group of major depressive disorder (**B**) and dysthymia (**D**) in China, 1990 to 2019

Figure 2 provides the age trend in MDD and dysthymia rate by different period, as well as the cohort trend by age group. Notably, the incidence rates of all six periods increased gradually with age, and the incidence rate for people in the 50−54 years and younger group decreased with period. The period of 2015–2019 had the greatest change in incidence rate: the lowest rate was observed at younger ages, while for the elderly the highest rate was found. The later birth cohort had a lower MDD incidence at younger ages, but their incidence was higher at older ages than the earlier birth cohort (Fig. 2A). There were the same trends in dysthymia incidence for six periods, and all have a peak at the age group of 40–44 (Fig. 2C). For MDD, before the 1950–1960 birth cohort, there was a short decreasing trend followed by a steer increasing trend with the birth cohort, while there was only decreasing trend after the birth cohort 1950–1960 with birth cohort (Fig. 2B). MDD had similar results with depressive disorders, whereas the rate of dysthymia had changed little with birth cohort (Fig. 2D).

### Joinpoint regression analysis

**Fig. 3:**
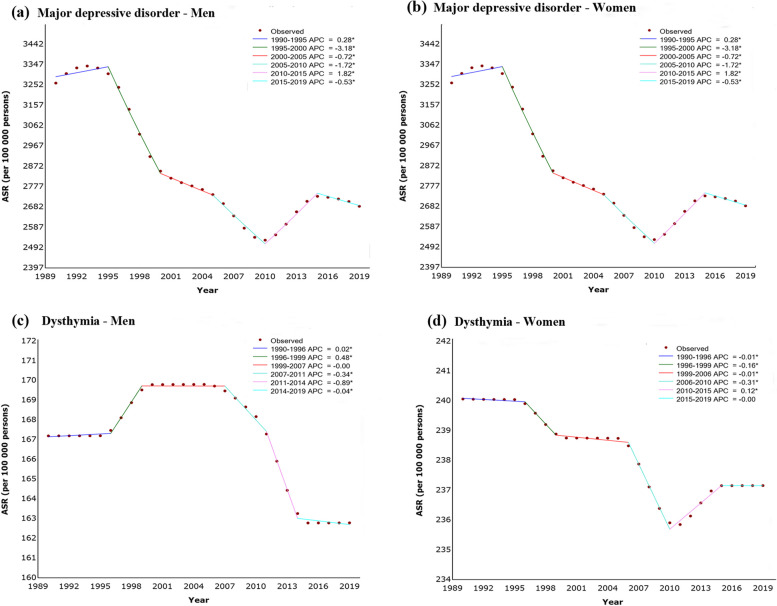
Joinpoint regression analysis in sex-specific age-standardized incidence rates of major depressive disorder and dysthymia in China from 1990 to 2019.  Notes: an asterisk indicates that the annual percent change is statistically significantly different from zero at the α = 0.05 level

The APCs (95% CI) of each segment for MDD and dysthymia are shown in Fig. 3 and their values are listed in Supplementary Table 2. The AAPC of MDD from 1990 to 2019 was -0.28 (-0.52, -0.03) in men, -0.69 (-0.79, -0.60) in women, and the dysthymia was − 0.09 (-0.10, -0.09) in men, -0.04 (-0.05, -0.04) in women, respectively. Consequently, results from joinpoint regression showed an overall decreasing trend in the rates of depressive disorders, including their subtypes. The AAPCs of gender-age-specific rates of depressive disorders are listed in Table 1, and the rates of MDD and dysthymia are listed in Supplementary Table 3. The results showed that young people (< 50 years) presented a declining trend, whereas the elderly (≥ 50 years) showed an increasing trend both in men and women for MDD and dysthymia.

**Table 1:** The gender-age-specific changes of depressive disorders in China from 1990–2019

	Man AAPC, 95% CI (%, 1990–2019)	Women AAPC, 95% CI (%, 1990–2019)
ASR	-0.26 (-0.46, -0.06)	-0.65 (-0.74, -0.56)
10–14 years	-0.19 (-0.35, -0.02)	-0.18 (-0.29, -0.06)
15–19 years	-1.00 (-1.38, -0.61)	-1.57 (-1.94, -1.21)
20–24 years	-1.36 (-1.63, -1.09)	-2.19 (-2.41, -1.97)
25–29 years	-1.38 (-1.67, -1.09)	-2.09 (-2.25, -1.94)
30–34 years	-1.30 (-1.56, -1.05)	-1.84 (-1.9, -1.78)
35–39 years	-1.20 (-1.41, -1.00)	-1.57 (-1.65, -1.49)
40–44 years	-0.76(-1.00, -0.52)	-1.05 (-1.13, -0.98)
45–49 years	-0.17 (-0.53, 0.19)	-0.32 (-0.47, -0.18)
50–54 years	0.35 (0.11, 0.60)	0.32 (0.16, 0.47)
55–59 years	0.82 (0.57, 1.07)	0.85 (0.67, 1.02)
60–64 years	1.01 (0.89, 1.13)	1.08 (0.88, 1.27)
65–69 years	0.97 (0.76, 1.18)	1.03 (0.86, 1.2)
70–74 years	0.96 (0.70, 1.21)	1.00 (0.83, 1.16)
75–79 years	0.91 (0.80, 1.01)	0.97 (0.81, 1.12)
80–84 years	0.75 (0.63, 0.87)	0.86 (0.72, 0.99)
85–89 years	0.56 (0.52, 0.60)	0.66 (0.61, 0.71)
90–94 years	0.40 (0.32, 0.48)	0.49 (0.43, 0.56)

### Age–period–cohort analysis

**Fig. 4:**
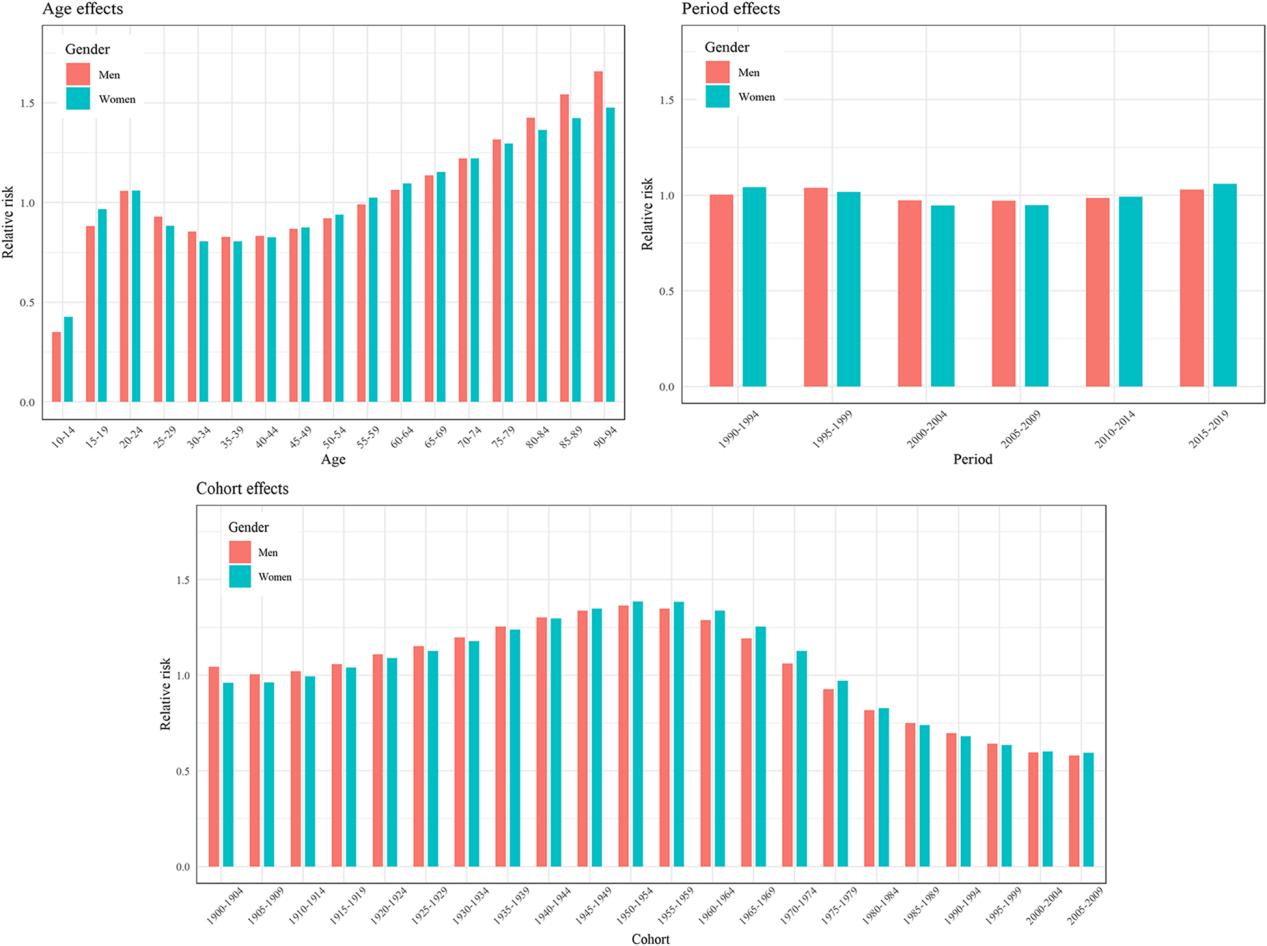
Relative risks of MDD in China from 1990 to 2019 due to age, period, and cohort effects

**Fig. 5:**
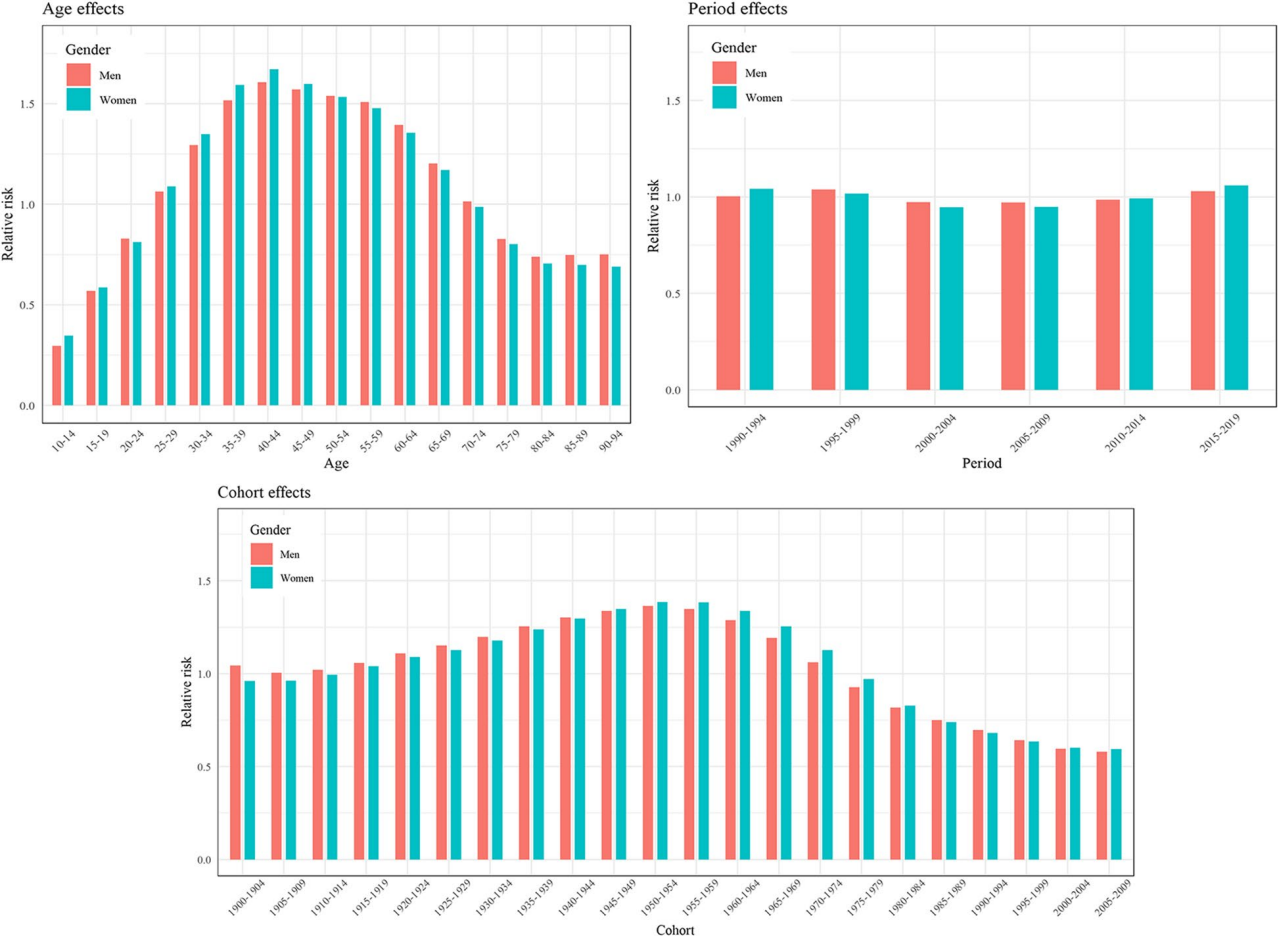
Relative risks of dysthymia in China from 1990 to 2019 due to age, period, and cohort effects

The RRs of depressive disorders estimated by the age–period–cohort model using the intrinsic estimator approach are illustrated in Figs. 4 and 5, and specific values of RRs can be found in Supplementary Table 3. Estimated RRs for age, period and cohort were obtained separately after controlling the effects of the other two factors. The RRs for age in MDD incidence had an overall increasing trend with age. Furthermore, when the population aged 75–79 and older, men have greater RRs than women, as well as a larger increase range (Fig. 4). The group aged 90–94 had the largest RR, 1.57 (95% CI: 1.55, 1.60) for men and 1.41 (95% CI: 1.39, 1.43) for women. Regarding period RRs, they initially decreased, then stabilized, and eventually increased for both genders, forming a U-shaped pattern (Fig. 4). The largest RR for period effect was 1.06 (95% CI: 1.05, 1.06) in period 2015–2019 for women. Cohort RRs presented an inverted U-shaped pattern, and with the highest peak of the cohort born in 1950–1954, RR = 1.33 (95% CI:1.31, 1.35) for men and RR = 1.39 (95% CI: 1.27, 1.40) for women. RRs in dysthymia for period and cohort effects had no statistical significance, only the age effect presented an inverted U-shaped pattern with the highest risks (RR = 1.61 (95%CI: 1.52, 1.69) for men, RR = 1.67 (95%CI: 1.6, 1.75) for women) in the group aged 40–44 (Fig. 5).

## Discussion

To our knowledge, the current study is the first to determine the secular incidence trend of MDD and dysthymia at the same time using the intrinsic estimator age-period-cohort framework. The findings indicated a decreasing trend in the Age-Standardized Rates (ASR) of MDD and dysthymia among both men and women over the past three decades. Joinpoint regression analysis further revealed that this declining trend primarily affected individuals under the age of 50, while an increasing trend was observed among the elderly (aged 50 and above) in both genders during the same period. Moreover, age-period-cohort models demonstrated that age was identified as the most influential factor for MDD and dysthymia, with age-related risks for MDD incidence exhibiting an overall increasing trend across different age groups. Additionally, MDD was found to be more susceptible to the effects of age, period, and cohort than dysthymia. Furthermore, the impact of age effects differed significantly between the two disorders.

Although the overall ASRs of depressive disorders and its subtypes have been decreasing in China during 1990–2019, the incidence number has been increasing. The increased number might be largely attributed to population growth and aging. Notably, women had a high incidence rate of depressive disorders than men, which aligned with previous studies [2, 34, 35]. A comprehensive review encompassing evidence from symptomology, treatment, pathophysiology and epidemiology suggested that differences in gene expression, neuroplasticity and immune might account for the discrepancy in the incidence rate of depression between men and women [36]. From the view of epidemiology, studies have shown that women were twice as likely as men to suffer sexual abuse, both in childhood and adulthood, and the prevalence of maternal depression have been also increasing dramatically in China [37]. These negative events would have a long-lasting and even lifelong adverse impact on women [37]. Therefore, it is crucial to provide women with enhanced medical care and social support, particularly during vulnerable periods such as childhood, menstruation, and pregnancy.

The first objective of this study was to determine the secular trend of depressive disorders in China. The total incidence of depressive disorders was not presented in our study due to dysthymia had a much small incidence compared with MDD. The reason for the lower prevalence of dysthymia compared to major depressive disorder (MDD) may be attributed to underreporting and underdiagnosis, as well as variations in diagnostic criteria and classification [7, 8]. From a global perspective, a study found that the incidence number of depression worldwide increased by 49.86% from 1990 to 2017, and the ASR increased significantly in well-developed regions and high-income North America [38]. There were studies suggesting that the increased ASR of world depression was associated with high levels of material lifestyle, education, and social pressure [39–41]. In addition, to our knowledge, there was very little published literature on changes of trends in dysthymia incidence in China. Our results may provide a little insight into this gap: the results of the current study suggested that the trend of dysthymia decreased in the group younger than 55 years old from 1990 to 2019 regardless gender, while the elderly group (older than 55 years old) continuously increased during the 30 years. The findings were consistent with previous studies [42, 43]. Several factors may explain this phenomenon. Firstly, developmental factors and life stage may contribute to the rising depression rates among older age groups [4]. Factors such as age-related health issues, chronic illnesses, social isolation and the challenges associated with aging may play a role. Secondly, socioeconomic and cultural changes may also be influential [13]. In recent years, there has been improved awareness, reduced stigma surrounding mental health and increased accessibility to mental health services. These factors may have contributed to the decreasing trend of depression in younger age groups. Lastly, cohort effects may contribute to the observed disparities. Older adults in the present era have experienced significant changes in China over the past 30 years. It is plausible that the earlier cohort, consisting of older adults, may have been exposed to more risk factors.

While MDD and dysthymia share similar symptoms and definitions, the latter had shown a smaller change than the former over the past 30 years. From the perspective of definition, MDD has a high severity with low chronicity while dysthymia is opposite. In general, dysthymia could manifest itself in elusive and long-lasting ways, resulting that it was considered as the more difficult of two to diagnose, and it is difficult to recognize in primary care until it aggravates in the form of an overlapped major depressive disorder episode. Therefore, the observed smaller change in dysthymia might as a result of the discrepancy between MDD and dysthymia. Importantly, the declines in the incidence rate of MDD have been slower in the past two decades. This slower decline could be attributed to factors such as rapid aging, the rapidly increasing economy and social pressure in China. A recent longitudinal study in China found a statistically significant association between the rapid growth of GDP per capita and a deterioration of mental health, mainly indicated by more serious depression symptoms and cognitive disorders in the 21st century [44].

Over the last three decades, we have observed a noticeable discrepancy in the prevalence of dysthymia between genders. Several factors could account for this disparity. One possible explanation lies in biological factors, including hormonal fluctuations, genetic predisposition, and variances in brain structure and function between males and females [34, 35]. These factors have the potential to impact the expression and susceptibility to dysthymia. Additionally, cultural and social factors have been recognized as significant contributors to the gender differences observed in depression [36].

Results from age-period-cohort models were helpful to understand the changes in the incidence trend of depressive disorders. The results showed that age played the most important role in the incidence of MDD and dysthymia. A cohort study based on a large population in Denmark found that age-specific incidence rates of mood disorders (defined by ICD-10) had a bimodal distribution in both genders, and the two peaks were in the early third and the ninth decades of life stages [45]. The results were different from the current study (Fig. 2A). The difference could be due to the cohort effects were taken into account in the age-period-cohort models, whereas the other study didn’t, and social pressures between Denmark and China could also contributed to the variation in results.

It was not surprising that the RRs for age in MDD continuously increased with age group, however, the RRs for age in dysthymia presented an interesting inverted U-shaped pattern, indicating that the risk was relatively lower among both the young and the elderly population. To our knowledge, there is no literature available that specifically addresses or offers an interpretation for these findings. It seems possible that these results are due to the following. The elderly population might experience an increase and accumulation of various risk factors that result in more severe depressive symptoms or episodes, which were then diagnosed as MDD. However, this speculation has yet to be confirmed by research and warrants further investigation. In the current study, it is not surprising to observe the first hazard RRs (RR > 1) for dysthymia in the 25–29 age group. This could partly be attributed to the fact that young adults who have recently completed their education and are transitioning into the workforce often experience uneasiness and stress related to societal expectations and job-seeking. These negative events can increase the risk of developing depressive symptoms [46].

The period RR represents the effects of outside factors at a particular calendar time. In this study, the RRs for MDD and dysthymia were all very close to one, suggesting stability in China from 1990 to 2019 without war, famine, or unrest. Furthermore, China’s social and economic development continued, and the material and spiritual life of the people was gradually enriched during the past 30 years. It is worth mentioning that the RRs of period were not consistent between men and women, possible reasons could be that in the past 30 years, China’s economy, sociocultural norms, and social structures have undergone significant changes, but these changes have not uniformly affected men and women [47, 48]. Specifically, during this period, Chinese women have experienced significant improvement in their economic status and independence, leading to greater participation in professional and social activities. However, they still face challenges of workplace gender discrimination, family responsibilities, and balancing career and family, whereas men have not experienced such drastic changes in their economic status and social responsibilities as women have.

Regarding cohort effects, MDD was found to be more affected compared to dysthymia. We determined that RRs for cohort effects in MDD increased from 1910 to 1914 cohort, reaching the highest at 1950–1954 cohort, and then gradually decreased to the lowest of 0.58 (0.53, 0.64). The hazard RR (RR > 1) persisted for about 70 years from 1910 to 1974. Those people who were born in an unrested time experienced abundant negative events, such as World War I (1914–1918), world war II (1939–1945) and the Chinese Civil War (1927–1949). These social, cultural and political disruptions had a substantial negative impact on social integration and cultural perceptions within Chinese society [49]. Subsequently, after the establishment of the People’s Republic of China in 1949, the cohort RR of MDD gradually declined. Despite a relatively stable environment after 1949, people in that period also experienced several historical events, including the Great Chinese Famine (1959–1961), the SARS pandemic (2002–2003) and the Wenchuan earthquake (2008) in the second half of their life course.

It is noteworthy that the cohort effect of 1960–1964 cohort in this period still exceeded 1(RR = 1.29, 95%CI: 1.26–1.31), indicating that the Great Famine continued to have a negative impact on the mental health of Chinese people, which was consistent with the results of other studies [50, 51]. In contrast, people born after 1964 experienced improved living conditions and more stable environments, resulting in a continuous decline in the RRs for cohort effects of MDD. Besides, China has formed many treatment methods for mental illness to cope with the difficulty of managing patients with severe mental illness [52]. Therefore, the continued attention and investment of the Chinese government could in a large extent account for the continuous decrease in the cohort effects as the birth cohort moved forward [52, 53].

The differences in incidence trends between MDD and dysthymia over the period 1990–2019 suggested that they might represent two distinct disease entities. Previous studies from follow-up, family and clinical treatment suggested that a strong relationship between dysthymia and MDD, thus supporting dysthymia as a mood disorder. However, there have been arguments asserting that they are two separate illnesses, and our current study aligned with this perspective. Our findings also revealed disparities in the incidence rate, cohort trends, period trends and age effects, further emphasizing the differing nature of these disorders.

The findings in this report are subject to at least three limitations. First, the data on depressive disorders from GBD 2019 were estimated, whose limitations were illustrated in the other literature [2]. Second, due to the design of the current study was an ecological study, and age-period-cohort models take a community as an analytical unit, so there might be ecological fallacies in the results, thus, we stress that the results of temporal trends in this study were based on the available data. Third, APC models of aggregated data are not a substitute for studies that use individual data to test research hypotheses. But, the results of such analyses, if conducted correctly, could provide useful data to support research with individual-level data. Additionally, due to insufficient data, we were unable to consider the effects of double depression (i.e., being diagnosed with both MDD and dysthymia) in our analyses. However, the purpose of this study was to separately analyze the individual trends of both conditions in order to identify potential differences, and our final results indicated the presence of such differences. Therefore, we believe that the conclusions drawn from this study remain reliable.

## Conclusion

The age, period and cohort effects all had a greater impact on MDD than on dysthymia, and age effects presented different influential patterns, which indicated that distinguishing these two diseases might be meaningful. From the perspective of historical epidemiology, the cohort effect had a higher risk before the cohort 1970–1974. A continuously increasing incidence number of depressive disorders was observed in China from 1990 to 2019 in both genders, indicating a potentially increasing burden in the future. Action should be taken to reduce the burden of depressive disorders in China, and the elderly need more attention.

### Supplementary Information

**Additional file 1:** **Supplementary Table 2.** Temporal trends of depressive disorders, major depressive disorders and dysthymia in China (1990-2019), results from the joinpoint regression analysis. **Supplementary Table 3.** The gender-age-specific rates of major depressive disorders and dysthymia in China in 2019 and the percentage changes from 1990-2019. **Supplementary Table 4. **Relative risks of major depressive disorders and dysthymia in China from 1990 to 2019 due to age, period, and cohort effects.

## Data Availability

Data sources used in this study can be obtained freely from the Institute for Health Metrics and Evaluation (https://vizhub.healthdata.org/gbd-results/).

## Abbreviations

MDD: Major depressive disorder
GBD: The global burden of disease study
RR: Relative risk
DALYs: Disability-adjusted life-years
DSM-IV-TR: Diagnostic and statistical manual of mental disorders fourth edition, text revision
UIs: Uncertainty intervals
APC: Annual percent change
AAPC: Average annual percent change
CI: Confidence interval
ASR: Age-standardized rates
