# Supplementary material for: Secular trends in the incidence of major depressive disorder and dysthymia in China from 1990 to 2019
Source: BMC Public Health. 2023 Nov 6;23:2162. doi: 10.1186/s12889-023-17025-4 (PMC10626640; doi:10.1186/s12889-023-17025-4)
Supplement: Supplementary file 1 — Additional file 1: Supplementary Table 2. Temporal trends of depressive disorders, major depressive disorders and dysthymia in China (1990-2019), results from the joinpoint regression analysis. Supplementary Table 3. The gender-age-specific rates of major depressive disorders and dysthymia in China in 2019 and the percentage changes from 1990-2019. Supplementary Table 4. Relative risks of major depressive disorders and dysthymia in China from 1990 to 2019 due to age, period, and cohort effects. [file 12889_2023_17025_MOESM1_ESM.docx]

**Supplementary Table 2.** Temporal trends of depressive disorders, major depressive disorders and dysthymia in China (1990-2019), results from the joinpoint regression analysis.

|  | **Depressive disorders** | | **Major depressive disorders** | | **Dysthymia** | |
| --- | --- | --- | --- | --- | --- | --- |
|  | **Men** | **Women** | **Men** | **Women** | **Men** | **Women** |
| **Trend 1** |  |  |  |  |  |  |
| Year | 1990-1992 | 1990-1995 | 1990-1992 | 1990-1995 | 1990-1996 | 1990-1996 |
| APC (95% CI) | 5.94 (4.41, 7.49) * | 0.26 (0.05, 0.46) * | 6.51 (4.68, 8.37) * | 0.28 (0.06, 0.5) * | 0.02 (0.01, 0.02) * | -0.01 (-0.01, 0) |
| **Trend 2** |  |  |  |  |  |  |
| Year | 1992-1995 | 1995-2000 | 1992-1995 | 1995-2000 | 1996-1999 | 1996-1999 |
| APC (95% CI) | 2.31 (0.86, 3.77) * | -2.96 (-3.22, -2.71) * | 2.52 (0.79, 4.28) * | -3.18 (-3.46, -2.91) * | 0.48 (0.44, 0.51) * | -0.16 (-0.18, -0.13) * |
| **Trend 3** |  |  |  |  |  |  |
| Year | 1995-2000 | 2000-2005 | 1995-2000 | 2000-2005 | 1999-2007 | 1999-2006 |
| APC (95% CI) | -3.04 (-3.45, -2.63) * | -0.66 (-0.91, -0.42) * | -3.34 (-3.83, -2.85) * | -0.72 (-1, -0.45) * | 0 (-0.01, 0) | -0.02 (-0.02, -0.01) * |
| **Trend 4** |  |  |  |  |  |  |
| Year | 2000-2005 | 2005-2010 | 2000-2005 | 2005-2010 | 2007-2011 | 2006-2010 |
| APC (95% CI) | -0.34 (-0.73, 0.06) | -1.6 (-1.84, -1.37) * | -0.37 (-0.85, 0.11) | -1.72 (-1.98, -1.47) * | -0.34 (-0.36, -0.32) * | -0.31 (-0.32, -0.29) * |
| **Trend 5** |  |  |  |  |  |  |
| Year | 2005-2009 | 2010-2015 | 2005-2009 | 2010-2015 | 2011-2014 | 2010-2015 |
| APC (95% CI) | -2.18 (-2.79, -1.58) * | 1.67 (1.42, 1.93) * | -2.39 (-3.13, -1.64) * | 1.82 (1.54, 2.09) * | -0.89 (-0.92, -0.85) * | 0.12 (0.12, 0.13) * |
| **Trend 6** |  |  |  |  |  |  |
| Year | 2009-2019 | 2015-2019 | 2009-2019 | 2015-2019 | 2014-2019 | 2015-2019 |
| APC (95% CI) | 0 (-0.10, 0.10) | -0.48 (-0.75, -0.22) * | 0.05 (-0.08, 0.17) | -0.53 (-0.81, -0.24) * | -0.04 (-0.05, -0.03) * | 0 (-0.01, 0.01) |

APC, annual percent change; CI, confidence interval.

* indicates the APC was significant different from zero at the alpha = 0.05 level.

**Supplementary Table 3.** The gender-age-specific rates of major depressive disorders and dysthymia in China in 2019 and the percentage changes from 1990-2019

|  | **Men** | | **Women** | |
| --- | --- | --- | --- | --- |
|  | Rates in 2019, 95% UI (per 100 000 population) | AAPC, 95% CI (%, 1990–2019) | Rates in 2019, 95% UI (per 100 000 population) | AAPC, 95% CI (%, 1990–2019) |
| **Major depressive disorders** |  |  |  |  |
| ASR | 1526.03 (1721.13, 1346.86) | -0.28 (-0.52, -0.03) | 2684.92 (3023.11, 2371.05) | -0.69 (-0.79, -0.60) |
| 10-14 years | 467.48(263.66, 734.84) | -0.18 (-0.37, 0.00) | 992.54(584.9, 1499.5) | -0.22 (-0.36, -0.08) |
| 15-19 years | 1167.62(834.08, 1588.48) | -1.04 (-1.44, -0.65) | 2176.8(1566.12, 2931.38) | -1.67 (-2.04, -1.29) |
| 20-24 years | 1494.98(1036.58, 2005.97) | -1.45 (-1.73, -1.17) | 2512.61(1734.87, 3390.49) | -2.32 (-2.54, -2.1) |
| 25-29 years | 1470.67(1061.47, 1986.6) | -1.51 (-1.8, -1.21) | 2387.35(1708.2, 3240.59) | -2.26 (-2.43, -2.08) |
| 30-34 years | 1482.2(1082.22, 1997.25) | -1.45 (-1.71, -1.19) | 2470.31(1788.85, 3354.27) | -2.02 (-2.09, -1.95) |
| 35-39 years | 1522.05(1109.98, 1958.37) | -1.36 (-1.58, -1.15) | 2765.96(1990.7, 3561.21) | -1.73 (-1.83, -1.62) |
| 40-44 years | 1747.62(1252.77, 2246.01) | -0.86 (-1.14, -0.58) | 3287.83(2391.65, 4217.42) | -1.15 (-1.24, -1.07) |
| 45-49 years | 2156.26(1714.31, 2629.24) | -0.19 (-0.6, 0.22) | 4018.08(3223.48, 4891.69) | -0.35 (-0.5, -0.19) |
| 50-54 years | 2574.66(2065.38, 3169.97) | 0.42 (0.18, 0.66) | 4739.36(3802.81, 5816.41) | 0.34 (0.18, 0.51) |
| 55-59 years | 2967.70(2312.3, 3748.85) | 0.91 (0.65, 1.16) | 5415.75(4189.41, 6774.39) | 0.91 (0.72, 1.09) |
| 60-64 years | 3263.00(2618.26, 4035.18) | 1.10 (0.97, 1.23) | 5816.77(4642.52, 7149.82) | 1.15 (0.93, 1.36) |
| 65-69 years | 3478.15(2903.66, 4156.82) | 1.04 (0.85, 1.24) | 5976.91(4991.37, 7100.68) | 1.08 (0.90, 1.26) |
| 70-74 years | 3696.06(2996.82, 4532.18) | 1.01 (0.76, 1.27) | 6150.93(5004.38, 7442.45) | 1.04 (0.87, 1.22) |
| 75-79 years | 3895.22(2811.58, 5013.86) | 0.97 (0.71, 1.23) | 6289.24(4649.89, 8028.8) | 1.00 (0.84, 1.17) |
| 80-84 years | 4097.62(3039.29, 5210.82) | 0.78 (0.66, 0.90) | 6360.68(4716.59, 8117.15) | 0.89 (0.74, 1.03) |
| 85-89 years | 4264.01(3329.72, 5309.39) | 0.58 (0.54, 0.62) | 6353.52(4952.32, 7886.25) | 0.68 (0.63, 0.73) |
| 90-94 years | 4474.64(3251.47, 5945.48) | 0.41 (0.32, 0.51) | 6343.85(4614.74, 8448.5) | 0.51 (0.44, 0.58) |
| **Dysthymia** |  |  |  |  |
| ASR | 162.79 (195.35, 133.35) | -0.09 (-0.10, -0.09) | 237.16 (286.37, 195.67) | -0.04 (-0.05, -0.04) |
| 10-14 years | 47.73(29.18, 72.55) | -0.22 (-0.23, -0.2) | 91.35(57.17, 136.39) | 0.31 (0.3, 0.32) |
| 15-19 years | 93.2(56.7, 142.07) | -0.3 (-0.3, -0.29) | 151.34(94.6, 222.28) | 0 (-0.02, 0.02) |
| 20-24 years | 141.37(89.77, 212.79) | -0.23 (-0.24, -0.22) | 206.81(133.53, 299.66) | -0.12 (-0.14, -0.11) |
| 25-29 years | 187.55(139.63, 242.87) | -0.18 (-0.18, -0.17) | 273.45(204.01, 358.68) | -0.15 (-0.16, -0.14) |
| 30-34 years | 233.34(169.25, 316.51) | -0.14 (-0.15, -0.14) | 339.7(249.86, 460.48) | -0.16 (-0.16, -0.15) |
| 35-39 years | 278.66(190.56, 397.68) | -0.12 (-0.13, -0.11) | 404.54(271.98, 574.35) | -0.17 (-0.17, -0.17) |
| 40-44 years | 298.29(206.77, 409.96) | -0.1 (-0.11, -0.09) | 431.25(300.78, 595.79) | -0.14 (-0.15, -0.13) |
| 45-49 years | 293.37(216.42, 379.94) | -0.06 (-0.07, -0.05) | 421.28(312.29, 547.32) | -0.06 (-0.07, -0.05) |
| 50-54 years | 288.88(216.3, 381.46) | -0.02 (-0.03, -0.01) | 411.93(311.87, 534.2) | 0.01 (0, 0.02) |
| 55-59 years | 284.34(183.7, 405.12) | 0.02 (-0.02, 0.07) | 402.46(269.99, 579.31) | 0.09 (0.09, 0.1) |
| 60-64 years | 265.11(166.85, 386.56) | 0.04 (0.03, 0.06) | 371.92(234.37, 545.95) | 0.13 (0.12, 0.13) |
| 65-69 years | 231.2(166.09, 317.57) | 0.04 (0.02, 0.06) | 321.16(227.83, 448.75) | 0.1 (0.1, 0.11) |
| 70-74 years | 197.62(144.86, 263.42) | 0.03 (0, 0.06) | 271.05(198.29, 354.44) | 0.08 (0.08, 0.08) |
| 75-79 years | 163.87(105.14, 233.04) | 0.03 (0.02, 0.03) | 220.36(141.9, 314.02) | 0.04 (0.04, 0.04) |
| 80-84 years | 148.28(91.17, 216.9) | 0.01 (0, 0.02) | 194.18(119.10, 280.99) | 0.01 (0, 0.03) |
| 85-89 years | 150.73(106.27, 215.28) | 0 (0, 0) | 192.49(132.53, 267.39) | 0.01 (0, 0.02) |
| 90-94 years | 152.92(99.64, 235.18) | -0.03 (-0.03, -0.02) | 190.34(122.90, 289.09) | 0 (0, 0) |

**Supplementary Table 4.** Relative risks of major depressive disorders and dysthymia in China from 1990 to 2019 due to age, period, and cohort effects.

|  | Men | | Women | |
| --- | --- | --- | --- | --- |
|  | MDD | Dysthymia | MDD | Dysthymia |
| **Age** |  |  |  |  |
| 10-14 years | 0.35 (0.34, 0.36) | 0.3 (0.26, 0.33) | 0.43 (0.42, 0.44) | 0.35 (0.32, 0.38) |
| 15-19 years | 0.88 (0.86, 0.9) | 0.57 (0.53, 0.62) | 0.97 (0.95, 0.98) | 0.59 (0.55, 0.63) |
| 20-24 years | 1.06 (1.04, 1.08) | 0.83 (0.78, 0.89) | 1.06 (1.04, 1.08) | 0.81 (0.77, 0.86) |
| 25-29 years | 0.93 (0.91, 0.95) | 1.06 (1, 1.13) | 0.88 (0.87, 0.9) | 1.09 (1.03, 1.15) |
| 30-34 years | 0.85 (0.84, 0.87) | 1.29 (1.22, 1.37) | 0.8 (0.79, 0.82) | 1.35 (1.29, 1.41) |
| 35-39 years | 0.83 (0.81, 0.84) | 1.52 (1.44, 1.6) | 0.81 (0.79, 0.82) | 1.59 (1.52, 1.67) |
| 40-44 years | 0.83 (0.82, 0.85) | 1.61 (1.52, 1.69) | 0.83 (0.82, 0.84) | 1.67 (1.6, 1.75) |
| 45-49 years | 0.87 (0.85, 0.88) | 1.57 (1.49, 1.66) | 0.88 (0.86, 0.89) | 1.60 (1.53, 1.67) |
| 50-54 years | 0.92 (0.91, 0.94) | 1.54 (1.46, 1.62) | 0.94 (0.93, 0.95) | 1.53 (1.47, 1.61) |
| 55-59 years | 0.99 (0.97, 1.01) | 1.51 (1.43, 1.59) | 1.02 (1.01, 1.04) | 1.48 (1.41, 1.55) |
| 60-64 years | 1.06 (1.05, 1.08) | 1.40 (1.32, 1.47) | 1.10 (1.08, 1.11) | 1.36 (1.29, 1.42) |
| 65-69 years | 1.14 (1.12, 1.15) | 1.20 (1.14, 1.27) | 1.15 (1.14, 1.17) | 1.17 (1.11, 1.23) |
| 70-74 years | 1.22 (1.2, 1.24) | 1.01 (0.96, 1.08) | 1.22 (1.21, 1.23) | 0.99 (0.94, 1.04) |
| 75-79 years | 1.32 (1.3, 1.34) | 0.83 (0.78, 0.88) | 1.30 (1.28, 1.31) | 0.80 (0.76, 0.85) |
| 80-84 years | 1.43 (1.41, 1.45) | 0.74 (0.69, 0.79) | 1.36 (1.35, 1.38) | 0.71 (0.67, 0.75) |
| 85-89 years | 1.54 (1.52, 1.57) | 0.75 (0.70, 0.80) | 1.42 (1.41, 1.44) | 0.70 (0.66, 0.74) |
| 90-94 years | 1.66 (1.63, 1.68) | 0.75 (0.70, 0.81) | 1.48 (1.46, 1.49) | 0.69 (0.65, 0.74) |
| **Period** |  |  |  |  |
| 1990-1994 | 1.00 (0.99, 1.01) | 0.97 (0.94, 1.00) | 1.04 (1.03, 1.05) | 0.99 (0.96, 1.01) |
| 1995-1999 | 1.04 (1.03, 1.05) | 0.99 (0.96, 1.02) | 1.02 (1.01, 1.02) | 0.99 (0.97, 1.02) |
| 2000-2004 | 0.97 (0.96, 0.98) | 1.01 (0.98, 1.04) | 0.95 (0.94, 0.95) | 1.00 (0.97, 1.03) |
| 2005-2009 | 0.97 (0.96, 0.98) | 1.02 (0.99, 1.05) | 0.95 (0.94, 0.95) | 1.00 (0.98, 1.03) |
| 2010-2014 | 0.99 (0.98, 0.99) | 1.01 (0.98, 1.04) | 0.99 (0.99, 1.00) | 1.00 (0.98, 1.03) |
| 2015-2019 | 1.03 (1.02, 1.04) | 1.00 (0.97, 1.04) | 1.04 (1.03, 1.05) | 1.01 (0.99, 1.04) |
| **cohort** |  |  |  |  |
| 1900-1904 | 1.04 (1.01, 1.08) | 1.11 (0.95, 1.3) | 0.96 (0.94, 0.99) | 1.06 (0.92, 1.21) |
| 1905-1909 | 1.00 (0.98, 1.03) | 1.09 (0.98, 1.22) | 0.96 (0.94, 0.98) | 1.05 (0.95, 1.16) |
| 1910-1914 | 1.02 (1, 1.04) | 1.07 (0.97, 1.17) | 0.99 (0.98, 1.01) | 1.04 (0.96, 1.13) |
| 1915-1919 | 1.06 (1.04, 1.08) | 1.05 (0.97, 1.14) | 1.04 (1.03, 1.05) | 1.03 (0.96, 1.11) |
| 1920-1924 | 1.11 (1.09, 1.13) | 1.05 (0.97, 1.12) | 1.09 (1.08, 1.1) | 1.03 (0.96, 1.09) |
| 1925-1929 | 1.15 (1.13, 1.17) | 1.05 (0.98, 1.12) | 1.13 (1.11, 1.14) | 1.02 (0.97, 1.08) |
| 1930-1934 | 1.20 (1.18, 1.22) | 1.04 (0.98, 1.11) | 1.18 (1.17, 1.19) | 1.02 (0.97, 1.08) |
| 1935-1939 | 1.25 (1.23, 1.27) | 1.04 (0.98, 1.11) | 1.24 (1.22, 1.25) | 1.02 (0.97, 1.08) |
| 1940-1944 | 1.30 (1.28, 1.32) | 1.05 (0.98, 1.11) | 1.3 (1.28, 1.31) | 1.03 (0.97, 1.08) |
| 1945-1949 | 1.34 (1.32, 1.36) | 1.04 (0.98, 1.11) | 1.35 (1.33, 1.37) | 1.03 (0.98, 1.08) |
| 1950-1954 | 1.36 (1.34, 1.39) | 1.03 (0.97, 1.1) | 1.39 (1.37, 1.4) | 1.03 (0.98, 1.08) |
| 1955-1959 | 1.35 (1.32, 1.37) | 1.02 (0.96, 1.08) | 1.38 (1.36, 1.4) | 1.02 (0.97, 1.07) |
| 1960-1964 | 1.29 (1.26, 1.31) | 1.01 (0.95, 1.07) | 1.34 (1.32, 1.36) | 1.01 (0.96, 1.06) |
| 1965-1969 | 1.19 (1.17, 1.22) | 0.99 (0.93, 1.05) | 1.25 (1.24, 1.27) | 1.00 (0.95, 1.05) |
| 1970-1974 | 1.06 (1.04, 1.08) | 0.98 (0.92, 1.05) | 1.13 (1.11, 1.14) | 0.98 (0.94, 1.04) |
| 1975-1979 | 0.93 (0.91, 0.95) | 0.97 (0.91, 1.04) | 0.97 (0.96, 0.99) | 0.96 (0.92, 1.02) |
| 1980-1984 | 0.82 (0.8, 0.83) | 0.96 (0.9, 1.03) | 0.83 (0.81, 0.84) | 0.94 (0.89, 0.99) |
| 1985-1989 | 0.75 (0.73, 0.77) | 0.95 (0.88, 1.02) | 0.74 (0.73, 0.75) | 0.93 (0.87, 0.99) |
| 1990-1994 | 0.70 (0.68, 0.72) | 0.93 (0.85, 1.01) | 0.68 (0.67, 0.7) | 0.92 (0.86, 1.00) |
| 1995-1999 | 0.64 (0.62, 0.66) | 0.90 (0.80, 1.00) | 0.63 (0.62, 0.65) | 0.94 (0.86, 1.03) |
| 2000-2004 | 0.6 (0.57, 0.62) | 0.86 (0.74, 1.01) | 0.6 (0.58, 0.62) | 0.97 (0.86, 1.09) |
| 2005-2009 | 0.58 (0.53, 0.64) | 0.85 (0.63, 1.14) | 0.59 (0.56, 0.63) | 0.98 (0.79, 1.21) |
